# Exploring Lower Limb Biomechanical Differences in Competitive Aerobics Athletes of Different Ability Levels During Rotational Jump Landings

**DOI:** 10.3390/bioengineering12030220

**Published:** 2025-02-21

**Authors:** Qincheng Ge, Datao Xu, Zanni Zhang, Julien S. Baker, Huiyu Zhou

**Affiliations:** 1 Faculty of Sports Science, Ningbo University, Ningbo 315211, China; geqincheng1@gmail.com (Q.G.); xudatao3@gmail.com (D.X.); zhangzanni@gmail.com (Z.Z.); jsbaker@hkbu.edu.hk (J.S.B.); 2 Faculty of Engineering, University of Pannonia, H-8201 Veszprem, Hungary

**Keywords:** competitive aerobics athletes, different ability levels, rotational jump landing, lower limb biomechanics, injuries

## Abstract

High-level (HL) and low-level (LL) competitive aerobics athletes demonstrate different landing patterns during rotational jump landings, resulting in differing risks of lower limb injuries. This research aimed to investigate biomechanical differences between different levels of competitive aerobics athletes during rotational jump landings. The subjects included 15 male HL athletes and 15 LL athletes. This study captured kinematics, kinetics, muscle activation, and muscle force data, calculating joint stiffness, energy dissipation, anterior tibial shear force (ATSF), and patellofemoral joint contact force (PTF). LL athletes demonstrated significantly greater ankle dorsiflexion, inversion, and internal rotation angles; knee abduction angle and moment, internal rotation angle and moment; and smaller ankle plantarflexion moment and knee flexion angle. They also showed lower calf muscle coactivation, PTF, joint stiffness at the knee and hip, and the energy dissipation of the ankle and lower limb; greater thigh muscle coactivation and ATSF. The results show that LL athletes exhibit poorer stability at the ankle and knee joints, with a higher risk of anterior cruciate ligament (ACL) and ankle inversion injuries during rotational jump landings. To lower these risks, LL athletes should increase the flexion angle of the knee, hip, and ankle plantarflexion during landing.

## 1. Introduction

Competitive aerobics is a skill-driven sport that combines flexibility, strength, and control, characterized by both technical difficulty and aesthetic appeal. As competition rules evolve, the complexity of technical movements in competitive aerobics is increasing significantly [1]. However, while the demands of high-difficulty movements drive the development of competitive aerobics, they also increase the risk of sports injuries. This has become a major challenge for gymnasts, particularly those in competitive aerobics, throughout their careers [2,3,4]. In competitive aerobics, difficult movements are divided into three major groups: Group A (floor difficulty), Group B (aerial difficulty), and Group C (standing difficulty), with Group B having the largest quantity and variety of movements but also resulting in the greatest impact load. Gonçalves et al. surveyed athletes competing in the 2022 World Aerobics Championships and found that lower extremity injuries were most prevalent, particularly in the ankle and knee joints [5]. A research study investigating lower extremity injuries in competitive aerobics athletes within collegiate athletic associations found that ankle and knee joint injuries accounted for 53% and 69% of total injuries during training and competition, respectively, with most injuries related to landing impacts [6]. Competitive aerobics athletes endure hundreds of landing impacts per week, leading to prolonged exposure of the lower extremity to high-impact environments [7], which greatly increases the odds of lower extremity injuries. Therefore, exploring the effects of landing impacts on lower limb injuries not only helps optimize athletic techniques but also offers insights to prevent injuries, thus improving athletes’ performance and career safety.

Jumping, including rotational jumping, is a fundamental human movement performed in various sports such as gymnastics, competitive aerobics, and the long jump. Rotational jumps, like those in ballet, martial arts, and the 360° straight-body jump in competitive aerobics, are crucial for increasing the difficulty of performances. Mattiussi et al. found in a study of ballet that movements such as tour jetés require dancers to generate rotational momentum while maintaining postural control, leading to similar lower limb loading patterns as in competitive aerobics [8]. Similarly, gymnasts performing twisting dismounts experience comparable knee and ankle loading during landings [9]. Zhang and Shimokochi et al. studied the biomechanical characteristics of the lower limbs during landing from a certain height, finding that the landing process after jumping involves high-frequency and high-intensity musculoskeletal impacts, which often lead to more severe lower limb injuries [10,11], such as ligament strains, Achilles tendon injuries, and fractures [12,13,14]. Compared to normal landing maneuvers, the impact force during rotational jump landings is significantly greater. However, research on rotational jumps is relatively scarce. The centrifugal force generated by rotation affects the knee and ankle joints, increasing their instability. This creates significant challenges for athletes. To prevent injuries, athletes must possess excellent muscular control, relying on precise muscle coordination and reactions to effectively absorb impact and maintain joint stability.

Athletes at varying skill levels exhibit different movement patterns during landing. HL athletes typically display superior movement patterns compared to LL athletes, especially in movement control, joint stability, movement efficiency, and injury prevention. Smith et al. found that LL athletes scored significantly lower on the Landing Error Scoring System (LESS) test than HL athletes, indicating that LL athletes are more likely to adopt higher-risk movement patterns [15]. In addition, numerous studies have demonstrated that greater levels of athleticism are closely connected with better physical well-being. HL athletes tend to have greater muscular strength, higher endurance, and faster recovery, all of which contribute to their superior performance in training and competition [16,17,18]. Wong et al. found that athletes with lower skill levels are at a high risk of injury when participating in higher levels of competition [19]. Similarly, Jacobson et al. observed a comparable phenomenon when studying outstanding female soccer players at different competitive levels [20]. Therefore, it is particularly important to customize training strategies and injury prevention measures for athletes at different levels.

The knee joint is one of the most vulnerable joints for competitive aerobics athletes during high-impact movements. As a critical point for energy absorption and transfer in the lower extremity kinetic chain, its structure is complex and subject to extremely high mechanical loads [21,22,23]. In recent years, considerable research has focused on anterior cruciate ligament (ACL) injuries due to their common occurrence among athletes [24,25,26]. ACL injuries are prevalent, and most of these injuries occur during exercise [27,28,29]. Early research has indicated that 70–90% of ACL injuries occur without direct contact [30], with such injuries commonly occurring during activities like landing, deceleration, and changes of direction [31]. In competitive aerobics, most difficult movements end with high jump landings, especially after a series of aerial actions like rotations or leaps, followed by an immediate stop. This greatly increases the risk of knee joint injuries. Previous studies have demonstrated that the anterior tibial shear force (ATSF) is identified as the main factor leading to ACL injuries, as it increases the forward translation of the tibia relative to the femur, placing excessive tensile strain on the ACL [32]. Additionally, excessive knee abduction moment has been identified as a high-risk factor for ACL injuries, which is often observed in non-contact ACL injury mechanisms [33]. Furthermore, internal rotation moment at the knee joint will aggravate ACL strain by increasing the torsional load on the ligament, particularly during high-impact landings [34]. When ATSF exceeds the knee joint’s load-bearing threshold, it overstretches the ACL, leading to injury [35]. Patellofemoral contact force (PTF) can be identified as an essential element that affects the steadiness of the knee joint. An increase in PTF suggests a larger contact surface or a more uniform force spread across the bones, helping to reduce strain on specific places and improving the knee joints’ stability [36].

In competitive aerobics, the 360° straight-body jump requires precise movement control during landing after completing a rotational jump. This places substantial demands on knee stability and impact absorption. Incorrect landing techniques may significantly increase the risk of knee and ankle injuries, which varies among athletes of different levels. While there have been studies on landing biomechanics in various sports, there is limited research specifically focused on rotational jumps in competitive aerobics. Most existing studies have examined general landing mechanics or focused on non-rotational jumps, which do not account for the unique challenges posed by rotational movements. Additionally, although many studies have examined lower extremity injuries in competitive aerobics athletes [2,3,4,37], few studies have investigated these injuries from the perspective of sports biomechanics across athletes of different levels. Therefore, this research was aimed at exploring the biomechanical differences in the lower extremity between competitive aerobics athletes of various levels during the 360° straight-body jump landing. We hypothesized that, compared to HL athletes, LL athletes would exhibit a smaller flexion angle and a larger abduction angle at the knee joint, along with a larger inversion angle at the ankle joint. Additionally, LL athletes would demonstrate greater ATSF and smaller PTF, as well as smaller lower limb energy dissipation and knee joint stiffness. The findings of this study can provide an important theoretical basis for the prevention of sports injuries and the optimization of technical movements. Coaches and athletes can adjust their training strategies in a targeted manner.

## 2. Materials and Methods

### 2.1. Participants

Based on previous research [38], the sample size for this research was calculated by G-Power software (version: 3.1.9.7; Henry University of Düsseldorf, Düsseldorf, Germany). We used an independent samples *t*-test, and the effect size was 0.8 (power: 0.8, significance level: 0.05). A total of 30 male competitive aerobics athletes were randomly selected from Ningbo University’s aerobics team, including 15 high-level athletes (age: 20 ± 1.83 years; height 177.25 ± 2.87 cm; body weight (BW): 68 ± 3.56 kg) and 15 low-level athletes (age: 20.8 ± 1.79 years; height: 170.8 ± 6.06 cm; body weight (BW): 65.3 ± 5.4 kg). A high-level athlete was an athlete with a technical level designation of Sportsman or National Division 1. A low-level athlete was defined as an athlete with a technical level designation of National Division 2 and below. They had at least five years of competitive aerobics experience and trained 3–4 times a week. The subject selection criteria were as follows: (1) Studies on lower extremity injuries, particularly those involving musculoskeletal recovery in athletes, suggest that most non-surgical injuries, such as mild-to-moderate ligament sprains and muscle strains, show significant functional recovery within 6–9 months, depending on rehabilitation protocols and training resumption strategies. Therefore, our participants did not have a record of serious lower extremity operation or any other variable that might influence the study during the past seven months [39]. (2) There were no other elements that might interfere with sports performance. Each participant was made aware of the research purpose, demands, and experimental steps before signing the informed consent. The ethics committee of Ningbo University approved this study. Approval was granted prior to participating in the experiment (Approval Number: TY2024038).

### 2.2. Experimental Protocol and Procedure

**Figure 1:**
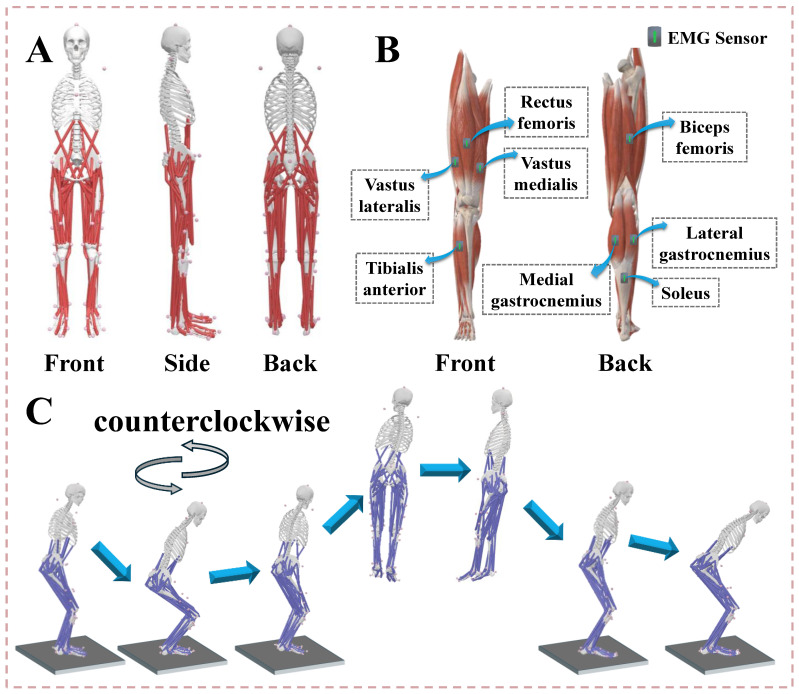
(**A**) Illustration of 38 reflective markers in three directions on a human musculoskeletal model. (**B**) Illustration of the position of EMG sensors on the muscles, including VL, VM, RF, BF, MG, LG, and SL. (**C**) Illustration of the complete movement of the 360° straight-body jump.

**Figure 2:**
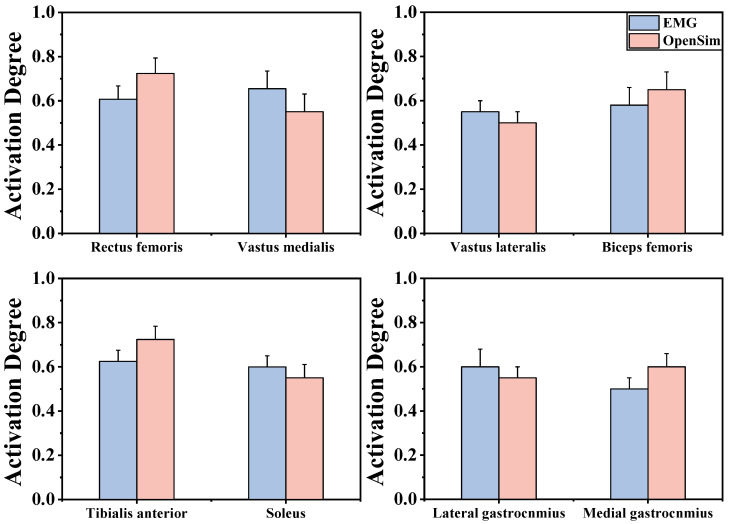
Illustration of the EMG/activation of muscles. The blue bar chart represents the result of EMG activation, and the red bar chart represents the result of OpenSim musculoskeletal modeling activation. The *x*-axis represents the muscle where the EMG sensor was placed. The *y*-axis represents the muscle activation degree, ranging from 0 to 1, where 0 indicates no activation and 1 indicates full activation.

Our experiments took place in the Exercise Biomechanics Laboratory of Ningbo University. Based on earlier research, the research team attached 38 standard reflective markers (with a diameter 12.5 mm) to subjects’ bodies to track their trajectories [40] (Figure 1A). An eight-infrared-camera Vicon Motion Capture System (Vicon Metrics Ltd., Oxford, UK) was utilized to record movement trajectories, using force plates (AMTI, Watertown, MA, USA) to acquire data of kinetics, with kinematics and dynamics sampled at frequencies of 200 and 1000 Hz [41]. Surface muscle activation and force data were collected using an electromyography (EMG) system, with a frequency of 1000 Hz [42] (Figure 1B). We placed the EMG sensors on the subjects’ vastus lateralis (VL), vastus medialis (VM), rectus femoris (RF), biceps femoris (BF), tibialis anterior (TA), medial gastrocnemius (MG), lateral gastrocnemius (LG), and soleus (SL). Maximum voluntary contractions (MVC) of these eight muscle groups were also collected to standardize muscle activation. Surface EMG was recorded after the test area was shaved, the skin was abraded, and alcohol was applied to minimize the impedance between the skin and electrodes [43]. The EMG results were compared to OpenSim-simulated muscle activation to validate the accuracy of the OpenSim musculoskeletal model. Figure 2 shows a good relevance between simulated muscle activation and EMG signals.

Before the formal experiment, the subjects wore uniform leggings and aerobics shoes. Participants ran on a treadmill for 10 min at a speed of 8 km/h as their warm-up, followed by structured muscle stretching exercises. To ensure consistency, all participants followed the same warm-up routine under the supervision of an experienced coach. To minimize potential fatigue effects, a five-minute resting period was provided after the warm-up before the experiment began. Then, the subjects practiced the entire test maneuver until they were familiar with the test procedure. During the familiarization phase, participants performed multiple practice trials and received standardized verbal instructions to ensure proper landing techniques. In the formal experiment, the subjects stood on the force plates in a standard anatomical position, arms extended diagonally downward at a 45° angle, and eyes looking forward [44]. Data were collected only from the dominant leg, which was defined as the preferred leg for kicking. Initial contact was defined as a ground reaction force greater than 10 N [45,46]. As shown in Figure 1C, the subjects stood at the center of the force plate. Upon hearing the command, they raised the arm on the side of their dominant leg, lifted the other arm sideways, and simultaneously jumped vertically with both feet. After completing a 360° straight-body jump, they landed on the ground with both feet together. A successful trial was defined if the subjects landed with both feet entirely on the force plate, remained upright without additional steps or foot adjustments, and maintained postural stability for at least two seconds after landing. A set of ten successful landing trials was collected for each subject. Between each session of the landing test, subjects rested for at least 30 s.

### 2.3. Data Collection and Processing

**Figure 3:**
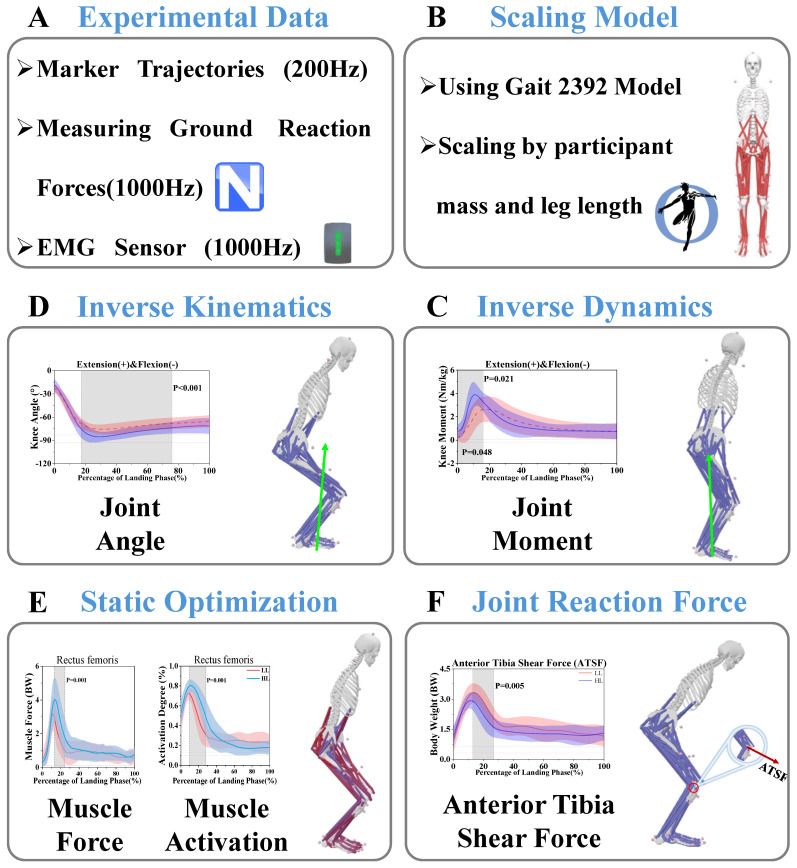
Process flow for musculoskeletal modeling to calculate muscle force, muscle activation, joint reaction force, etc. (**A**) Loading experimental data into OpenSim for analysis. (**B**) Scaling model of the subjects. (**C**) Inverse dynamics, calculating the joint moment, etc. (**D**) Inverse kinematics, calculating the joint angle, etc. (**E**) Static optimization, calculating the muscle force and activation. (**F**) Joint reaction force, calculating anterior tibia shear force (ATSF).

We used Vicon Nexus to collect the kinematic and dynamic data and export them to C3D format files [47]. Subsequently, we used MATLAB R2024a (MathWorks, Natick, MA, USA) to process experimental data, including procedures like coordinate conversion and low-pass filtering. We used the fourth-order zero-phase lag Butterworth low-pass filter to filter the data of kinematics and dynamics, and the cutoff frequencies were set to 10 Hz and 20 Hz [42,48]. Specifically, a 10 Hz cutoff frequency was used for kinematic data to effectively smooth marker trajectory data while preserving meaningful motion details, and a 20 Hz cutoff frequency was applied to dynamic data to minimize noise while maintaining the integrity of ground reaction force signals. Afterward, we converted the processed data into the trc format, which is necessary for OpenSim simulation software. Biomechanical parameters were processed and calculated by OpenSim in this study. The static model was imported into OpenSim 4.5 software and scaled to the subject’s model based on the Gait 2392 model, which had 23 degrees of freedom and 92 muscle actuators (Figure 3). Kinematic and kinetic data were computed by inverse kinematics (IK) and inverse dynamics (ID) in OpenSim 4.5.

Using a band-pass Butterworth filter to process the electromyography (EMG) signals, the cut-off frequencies were set to 10 Hz and 400 Hz [49]. To extract the EMG envelope, we applied a low-pass Butterworth filter to smooth and correct the signals, setting the cut-off frequency to 6 Hz [46]. Each muscle’s EMG envelope for each muscle was then normalized to peak. The maximal voluntary contraction (MVC) values were determined through three repeated trials of standardized maximal isometric contractions for each target muscle, each lasting 5 s against manual resistance. The highest root mean square (RMS) value among these trials was used as the normalization reference. To assess muscle activation, the EMG signal amplitude was divided by the maximum root mean square (RMS) value, which was then further normalized using this MVC-derived value [43].

Joint work was determined by integrating the joint power over time. To calculate the energy dissipation for each joint, the segments of the joint power curves that were negative were intercepted in this study. Negative (eccentric) work reflected the joint muscles’ energy dissipation [41]. To calculate the total energy dissipation in the lower extremity, the energy dissipation values of the ankle, knee, and hip joints were summed. In addition, dividing the energy dissipation value of each joint by the total energy dissipation value gave the percentage contribution of each joint’s energy dissipation to the total lower limb energy dissipation. The knee reaction forces calculated in OpenSim were transformed into the tibial reference frame and decomposed into anterior tibial shear forces (ATSF) [50,51]. Every parameter was calculated in MATLAB (MathWorks, Natick, MA, USA).

To calculate the coactivation of the muscles of the lower limb, the following equations were applied in this study [48]:

(1) $$\mathrm{Muscle}\;\mathrm{coactivation}\left(\%\right)=\left(\frac{{\mathrm{RMSEMG}}_{\mathrm{antagonist}}}{{\mathrm{RMSEMG}}_{\mathrm{agonist}}}\right)\times 100$$

${\mathrm{RMSEMG}}_{\mathrm{antagonist}}$ represents the root mean square error (RMSE) of muscle activation for antagonist muscles, and conversely, ${\mathrm{RMSEMG}}_{\mathrm{agonist}}$ represents the root mean square error (RMSE) of muscle activation for agonist muscles.

Our study calculated the joint stiffness of three joints (ankle, knee, and hip joint). We divided the variation of joint moment (ΔM) by the variation of joint angle (Δθ) to calculate the joint stiffness. Based on previous studies, we used $k_{\mathrm{joint}}=\frac{\Delta M}{\Delta \theta }$ to calculate the joint stiffness in this study [52].

By drawing on previous studies, the relationship between the patellofemoral joint contact force (PTF) during landing and the flexion angle (x) and extension moment (Mk) of the knee joint can be obtained [53]. At first, a nonlinear equation was utilized to calculate the force arm of the quadriceps muscle (Lq) through the flexion angle of the sagittal axis of the knee.

(2) $$\mathrm{Lq}=0.00008x^{3}\;-0.013x^{2}+0.28x+0.046$$

Then, we calculated quadriceps force (Fq) by the following formula:

(3) $$\mathrm{Fq}=\frac{\mathrm{Mk}}{\mathrm{Lq}}$$

Finally, we used the quadriceps force (Fq) and a constant (k) to compute PTF, and the non-linear equation described by van Eijden et al. was used to calculate the constant k for the position (x) of the knee joint angle. The computational formulas are as follows [54]:

(4) PTF=Fq × k 

(5) $$K=\frac{(0.462+0.00147x^{2}-\;0.0000384x^{2})}{(1\;-\;0.0162x^{2}+0.000155x^{2}\;-\;0.000000698x^{3})}$$

### 2.4. Statistical Analysis

Before performing statistical analysis, the dataset was first examined for normality using the Shapiro–Wilk test to assess whether the data followed a normal distribution. If the results did not satisfy the assumptions of normality, the Wilcoxon signed-rank test was applied for nonparametric data. For statistical parameter mapping (SPM) analysis, the entire dataset was extracted during the rotational jump landing. Then, the data points were interpolated by a custom MATLAB script, creating a 101 data point time-series curve, corresponding to 0% to 100% of the rotational jump lading. We performed statistical analysis using the open-source SPM1d script, setting a significance threshold of 0.05. Differences in peak variables between LL and HL athletes were calculated using an independent samples *t*-test in SPSS 27.0 for Windows software (Chicago, IL, USA), and the threshold of significance was 0.05 (*p* < 0.05).

## 3. Results

### 3.1. Joint Angle and Moment

**Figure 4:**
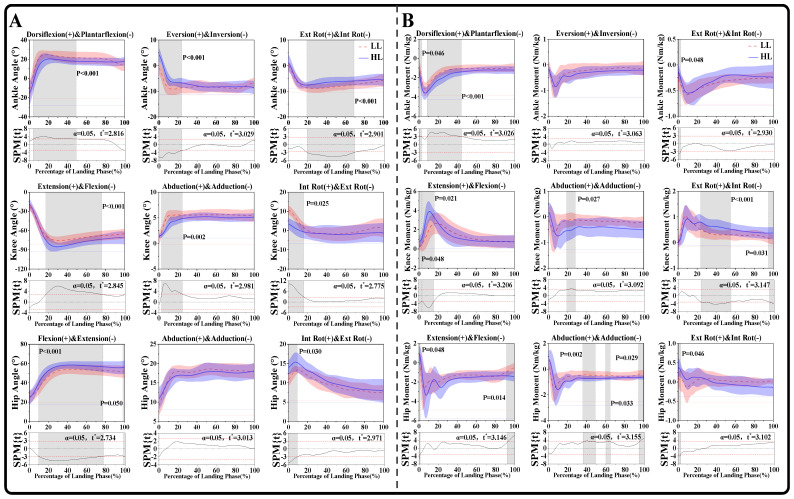
(**A**) Illustration of the statistical parametric mapping (SPM) results between LL and HL athletes during the landing phase of a 360° straight-body jump, depicting the angle changes of the ankle, knee and hip joints in the sagittal, frontal, and horizontal planes. The *x*-axis represents the percentage of the landing phase (%), ranging from 0% to 100%. The *y*-axis represents the joint angle (°). (**B**) Illustration of the statistical parametric mapping (SPM) results between LL and HL athletes during the landing phase of a 360° straight-body jump, depicting the moment changes of the ankle, knee and hip joints in the sagittal, frontal, and horizontal planes. The *x*-axis represents the percentage of the landing phase (%), ranging from 0% to 100%. The *y*-axis represents the joint moment (N·m/Kg). “t*” represents the threshold t-value. Gray-shaded areas indicate significant differences (*p* < 0.05) between LL and HL during the landing phase. Ext Rot: external rotation; Int Rot: internal rotation.

As shown in Figure 4A, there were significant differences in lower limb joint angles between LL and HL athletes. Specifically, at the ankle joint, LL athletes exhibited a significantly greater dorsiflexion angle, with differences ranging from 4.47% to 48.9% (*p* < 0.001). Additionally, LL athletes demonstrated a significantly greater inversion angle, with differences ranging from 0% to 24% (*p* < 0.001), and a notably greater internal rotation angle, with differences ranging from 19.4% to 69% (*p* < 0.001). At the knee joint, LL athletes showed a significantly smaller flexion angle, with differences ranging from 17.4% to 76% (*p* < 0.001), a markedly larger abduction angle, with differences ranging from 2.84% to 24.58% (*p* = 0.002), and a significantly larger internal rotation angle, with differences ranging from 0% to 16.13% (*p* = 0.025). Furthermore, at the hip joint, LL athletes exhibited a significantly smaller flexion angle, with differences ranging from 9.7% to 77.4% (*p* < 0.001) and from 98.36% to 100% (*p* = 0.050). LL athletes also demonstrated a smaller internal rotation angle, with differences ranging from 0% to 9.2% (*p* = 0.030). However, no significant differences were observed in the frontal plane of the hip joint, suggesting that athletes of different levels exhibit similar control and loading patterns in this plane during landing. This may indicate that frontal plane hip mechanics were less influenced by skill level compared to sagittal and transverse plane movements, where significant differences were found.

As presented in Figure 4B, there were significant differences in lower limb joint moments. The SPM analysis revealed that compared to LL athletes, HL athletes exhibited a significantly greater ankle plantarflexion moment, with differences ranging from 0% to 2.77% (*p* = 0.046) and from 8.56% to 44.38% (*p* < 0.001), and a smaller ankle internal rotation moment, with differences ranging from 0% to 2.58% (*p* = 0.048). However, no significant differences were observed in the frontal plane of the ankle joint, indicating that both HL and LL athletes exhibited similar movement patterns and loading characteristics in this plane during landing. This suggests that frontal plane ankle mechanics may not be a primary factor distinguishing athletes of different skill levels in rotational jump landing. Instead, the observed differences in other planes, such as sagittal and transverse, may play a more critical role in improving movement efficiency and preventing injury risk. Regarding the knee joint, HL athletes exhibited a significantly greater extension moment, with differences ranging from 0% to 2.79% (*p* = 0.048) and from 3.19% to 15.34% (*p* = 0.021), as well as a significantly greater adduction moment, with differences ranging from 19.52% to 27.45% (*p* = 0.027). Additionally, HL showed a significantly larger external rotation moment, with differences ranging from 23.7% to 54% (*p* < 0.001) and from 94.93% to 100% (*p* = 0.031). At the hip joint, HL athletes demonstrated a significantly greater flexion moment, with differences ranging from 0% to 1.24% (*p* = 0.048) and from 92.47% to 100% (*p* = 0.014), as well as a larger adduction moment, with differences ranging from 36.3% to 49% (*p* = 0.002), from 60.05% to 64.7% (*p* = 0.033), and from 94.59% to 100% (*p* = 0.029). Lastly, HL exhibited a significantly greater external rotation moment, with differences ranging from 0% to 2.75% (*p* = 0.046).

### 3.2. The Peak Angle, Moment, and Power

Table S1 illustrates the peak angle, moment, and power of three joints across three planes (sagittal plane, frontal plane, and transverse plane) between LL and HL athletes. First, at the ankle joint, LL athletes demonstrated significantly greater peak dorsiflexion angle and moment (*p* < 0.001; *p* < 0.001), internal rotation angle (*p* < 0.001), and eversion moment (*p* < 0.001) than HL athletes, as well as significantly smaller peak external rotation angle (*p* = 0.04), eversion angle (*p* < 0.001), external rotation (*p* < 0.001), and plantarflexion moment (*p* = 0.016). However, no significant differences were observed in terms of the peak power of the ankle joint, suggesting that both HL and LL athletes generated and absorbed energy at a similar rate during landing. This suggests that the peak power of the ankle joint may not be a key factor differentiating skill levels in rotational jump landing.

At the knee joint, LL athletes only exhibited significantly larger peak internal rotation (*p* < 0.001) and abduction angle (*p* < 0.001), along with significantly smaller peak extension angle (*p* = 0.026), flexion angle (*p* < 0.001), and flexion moment (*p* < 0.001) compared to HL athletes. Additionally, no significant differences were found in the peak values of other variables. This may indicate that these variables were less influenced by skill level or by other factors.

In terms of the hip joint, LL athletes demonstrated significantly smaller peak flexion and extension angle (*p* < 0.001, *p* = 0.627), internal rotation angle (*p* < 0.001), extension moment (*p* < 0.001), abduction moment (*p* = 0.024), and external rotation moment (*p* = 0.018) compared to HL athletes. Furthermore, no significant differences were observed in the peak values of other variables. Detailed results for peak values between the HL and LL athletes are provided in Supplemental Table S1.

### 3.3. Joint Energy and Joint Stiffness

**Figure 5:**
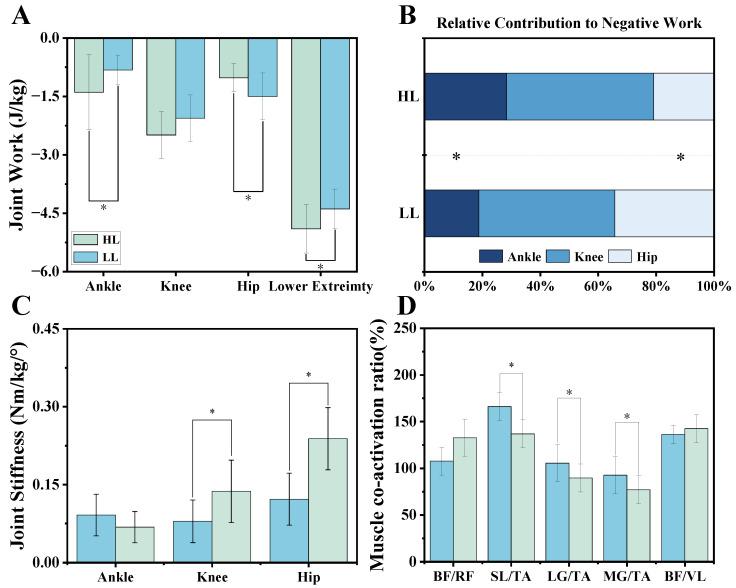
(**A**) Illustration of the mean energy dissipation of the lower limb joints and total lower extremity energy dissipation in the sagittal plane between LL and HL athletes. The *x*-axis represents the lower limb joints and the entire lower limb. The *y*-axis represents joint work (J/Kg). (**B**) Illustration of the respective contribution of lower limb joints to total energy dissipation. The *x*-axis represents the contribution to total energy dissipation, ranging from 0% to 100%. The *y*-axis represents HL and LL athletes. (**C**) Illustration of joint stiffness at each lower limb joint during the landing phase for LL and HL athletes. The *x*-axis represents the lower limb joints. The *y*-axis represents joint stiffness (N·m/Kg/°). (**D**) Illustration of muscle coactivation results in the lower limb for LL and HL athletes. The *x*-axis represents the muscles used to calculate coactivation, including five groups in total. The *y*-axis represents the muscle coactivation ratio (%). BF: biceps femoris; RF: rectus femoris; SL: soleus; TA: tibialis anterior; VL: vastus lateralis. “*” indicates a significant difference between HL and LL athletes (*p* < 0.05).

We found significant differences in lower limb energy dissipation between LL and HL athletes in the sagittal plane, specifically at the ankle, hip joint, and total lower limb energy dissipation (Figure 5A). However, no difference was found at the knee joint, suggesting that both HL and LL athletes exhibited similar energy dissipation mechanisms at this joint during landing. This may indicate that the knee played a more consistent role in absorbing impact forces regardless of skill level. Furthermore, significant differences were observed regarding the contribution of the ankle and hip joint to total energy dissipation (Figure 5B). In the sagittal plane, mean energy dissipation and contribution to total energy dissipation are shown in Table S2. Detailed results for mean energy dissipation and contribution to total energy dissipation between the HL and LL athletes are provided in Supplemental Table S2.

HL athletes, compared to LL athletes, exhibited significantly greater joint stiffness in the knee and hip joints. However, there was no significant difference at the ankle joint (Figure 5C).

### 3.4. Muscle Activation and Force

**Figure 6:**
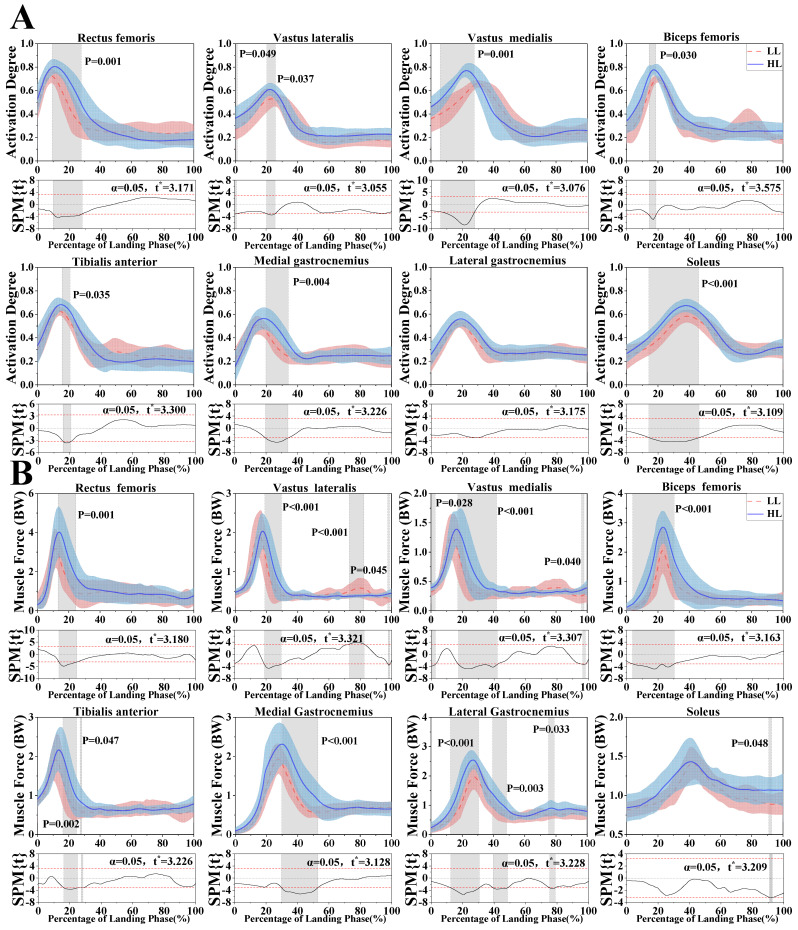
(**A**) Illustration of the mean and standard deviation of muscle activation in the lower limb muscles of LL and HL athletes during the landing phase, including RF, VL, VM, BF, TA, MG, LG, and SL. The *x*-axis represents the percentage of the landing phase (%), ranging from 0% to 100%. The *y*-axis represents the activation degree, ranging from 0 to 1, where 0 represents no activation, and 1 represents full activation. (**B**) Illustration of the mean and standard deviation of muscle force in the lower limb of LL and HL athletes during the landing phase, including RF, VL, VM, BF, TA, MG, LG, and SL. The *x*-axis represents the percentage of the landing phase (%), ranging from 0% to 100%. The *y*-axis represents muscle force. “t*” represents the threshold t-value. The shaded portion of the graphs indicates that there is a significant difference (*p* < 0.05) in the degree of muscle activation of LL and HL athletes.

Based on the results from the SPM analysis in Figure 6A, there were significant differences in muscle activation. These differences were found at different phases of activation. Specifically, significant differences were noted in the RF (9.5–27.84%, *p* = 0.001), TA (15.81–20.78%, *p* = 0.035), and MG (19.67–33.95%, *p* = 0.004) after and during the peak muscle activation. In addition, significant differences in muscle activation were observed in the VM (6.19–27.65%, *p* = 0.001), BF (14.35–18.61%, *p* = 0.030), and SOL (14.22–46%, *p* < 0.001) prior to and at the time of peak activation. Conversely, the VL showed significant differences only at the peak of muscle activation (0–1.29%, *p* = 0.049; 20.31–25.75%, *p* = 0.037). Additionally, no significant differences were observed in the LG, suggesting that its activation pattern remained consistent between HL and LL athletes during landing. This may indicate that the lateral gastrocnemius played a more stable role in lower limb muscle coordination, regardless of skill level. In contrast, the observed differences in other muscles highlighted variations in neuromuscular control strategies between the two groups, which could influence landing mechanics and impact absorption.

Figure 6B illustrates significant differences in muscle force between LL and HL athletes across various lower limb muscles during different landing phases. Specifically, HL athletes showed significantly greater force in the RF (13.17–24.54%, *p* = 0.001), and the VL in HL athletes exhibited higher force at multiple phases (18.97–29.62%, *p* < 0.001; 97.64–98.74%, *p* = 0.045). Furthermore, there were significant differences in the VM, with HL athletes showing higher force (0–2.67%, *p* = 0.028; 17.31–42.05%, *p* < 0.001; 96.32–98.02%, *p* = 0.040). The BF also showed a greater muscle force in HL athletes (3.81–30.35%, *p* < 0.001). The TA exhibited significantly greater force in HL athletes (16.32–25%, *p* = 0.002; 27.28–28.42%, *p* = 0.047). Similarly, compared to LL athletes, HL athletes exhibited significantly higher force in the MG (29.79–53%, *p* < 0.001), LG (12.44–30.54%, *p* < 0.001; 39.42–48.46%, *p* = 0.003; 75.29–78.87%, *p* = 0.033), and SL (90.91–92.68%, *p* = 0.048). Conversely, the VL of LL athletes demonstrated significantly greater force (72.91–82.39%, *p* < 0.001). These findings suggest that HL athletes generate higher muscle forces, particularly in key lower limb muscles, which are likely to contribute to better energy dissipation, joint stability, and overall landing performance compared to LL athletes.

Figure 5D shows that the muscle coactivation ratio was higher for HL athletes compared to LL athletes in the SL/TA, LG/TA, and MG/TA. Conversely, LL athletes exhibited a greater coactivation ratio than HL athletes in the BF/RF and BF/VL. These findings suggested that HL athletes displayed higher coactivation in the calf muscles, whereas LL athletes showed relatively higher coactivation in the thigh muscles.

Additionally, in Table S3, we observed significant differences in peak muscle activation between LL and HL athletes for the RF (*p* = 0.002), VM (*p* < 0.001), BF (*p* < 0.001), VL (*p* = 0.003), SL (*p* < 0.001), and TA (*p* = 0.015). Meanwhile, significant differences in peak muscle force were found between LL and HL athletes for the RF (*p* = 0.011), BF (*p* = 0.005), MG (*p* = 0.014), LG (*p* < 0.001), and VL (*p* = 0.037) (Table S3). Detailed results for peak values of muscle activation and force between HL and LL athletes are provided in Supplemental Table S3.

### 3.5. Anterior Tibia Shear Force and Patellofemoral Joint Contact Force

**Figure 7:**
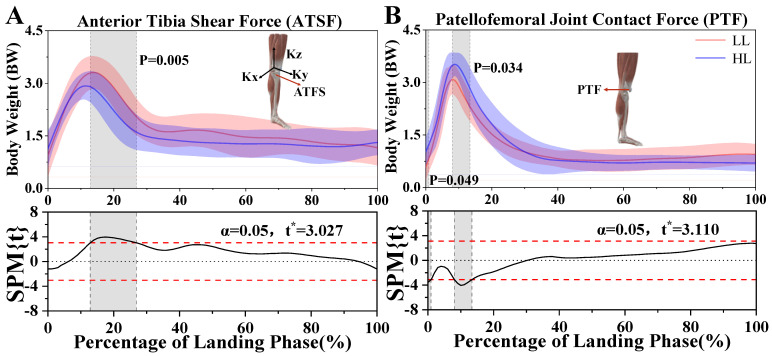
(**A**) Illustration of the statistical parametric mapping (SPM) results of the changes of ATSF in LL and HL athletes during the landing phase of a 360° straight-body jump. (**B**) Illustration of the statistical parametric mapping (SPM) results of the changes of PTF in LL and HL athletes during the landing phase of a 360° straight-body jump. The *x*-axis represents the percentage of the landing phase (%), ranging from 0% to 100%. The *y*-axis represents body weight (BW). “t*” represents the threshold t-value. The shaded portion of the graphs indicates that there is a significant difference (*p* < 0.05) in ATSF between HL and LL athletes.

Figure 7A shows a significant difference in ATSF between LL and HL athletes. The ATSF of LL athletes was significantly greater than HL athletes, ranging from 12.87% to 26.86% (*p* = 0.005) during the rotational jump landing. However, the PTF of HL athletes was significantly greater than LL athletes, ranging from 0% to 0.94% (*p* = 0.049) and from 8.06% to 13.39% (*p* = 0.034) (Figure 7B).

## 4. Discussion

The aim of this study was to explore any differences in lower extremity biomechanics during rotational jump landings between competitive aerobics athletes of different levels. The results supported the hypotheses, revealing significant differences between LL and HL athletes across multiple metrics. HL athletes demonstrated greater energy dissipation in the lower extremity, particularly at the ankle and hip joint, as well as higher joint stiffness at the knee and hip joint compared to LL athletes. LL athletes, on the other hand, showed a greater abduction angle, a smaller flexion angle of the knee joint, as well as a greater inversion angle of the ankle joint. Additionally, at the knee joint, HL athletes exhibited a significantly smaller ATSF and larger PTF than LL athletes. These findings suggest that LL athletes may be more susceptible to knee injuries and ankle injuries compared to HL athletes. HL athletes demonstrate superior stability in both the ankle and knee joints.

Several previous studies have investigated lower limb biomechanics during landing tasks in related fields like gymnastics, where similar patterns of joint angle have been observed [55,56,57]. A smaller knee flexion angle may increase ATSF, as well as the moment of internal rotation and abduction, which increases the likelihood of knee injuries, especially ACL injuries [58,59]. This finding aligns with studies by Xu et al., who reported that a smaller knee flexion angle during landing exhibited higher ATSF and a greater risk of ACL injuries [51]. A smaller knee flexion angle during landing may lead to insufficient shock absorption, resulting in higher anterior shear force at the knee joint. This places increased stress on the ACL, as it plays a more significant role in stabilizing the knee joint during impact. The reduced knee flexion prevents the proper dissipation of force, thereby increasing strain on the ACL and heightening the risk of injury. In our study, we found that LL athletes displayed significantly smaller knee flexion angles. Additionally, LL athletes exhibited a significantly larger knee abduction and internal rotation moment during the landing phase. These findings align with earlier research results, suggesting that LL athletes are more prone to ACL injuries compared to HL athletes. Excessive knee abduction is widely regarded as a dangerous movement pattern [33], and ACL injuries are frequently associated with the extent of knee abduction angle at the time of injury [25,60]. Our findings further confirm this suggestion. Furthermore, we observed LL athletes had a significantly smaller hip flexion angle compared to HL athletes, implying that LL athletes are less effective at absorbing ground reaction forces at the hip joint. This resulted in higher stress on the knee joint, substantially raising the likelihood of knee injuries.

In this study, LL athletes exhibited greater anterior tibial shear force (ATSF), suggesting that they experienced greater anterior shear forces at the knee joint during the landing task. Yu, Bing et al. found that ATSF is a primary factor contributing to ACL injuries. Similarly, Hume et al. found that ACL injuries accounted for 4.9% of sports injuries in gymnasts and that ATSF was a risk factor for ACL injuries [57]. The main function of the ACL is to limit the anterior movement of the tibia relative to the femur. When ATSF exceeds the knee joint’s threshold capacity, the ACL undergoes excessive tensile strain, which may lead to a tear [35]. An increase in ATSF typically indicates overloading of the knee joint, particularly during high-impact and high-speed movements, which increases the risk of ACL injuries [32]. HL athletes exhibited more precise movement control and lower anterior tibial shear force (ATSF) during landing, likely due to optimized lower extremity coordination and effective landing mechanics during training. Their superior technique enhances shock absorption, reducing the shear forces on the knee joint and lowering the risk of ACL injuries. This is also closely related to the knee flexion angle, as LL athletes displayed a smaller knee flexion angle during landing, which hindered the effective absorption of impact forces. Consequently, the increased ATSF in LL athletes further elevated the risk of ACL injuries.

The knee joint is essential for body balance [43,48], while patellofemoral contact force (PTF) plays a critical role in knee stability [61]. The patella acts as a mechanical pulley, improving the efficiency of knee extension by enhancing the lever arm of the quadriceps tendon [62]. The patellofemoral joint is important in activities that require substantial quadriceps activation, such as stair climbing or transitioning from sitting to standing [63]. Besier and Fitzpatrick have found that higher PTF increases the contact area between the patella and femur, improving force distribution and knee stability [64,65]. Our results indicate that HL athletes generated significantly higher PTF during landing compared to LL athletes, suggesting better control of the knee joint. Additionally, we found HL athletes exhibited greater knee joint stiffness. Joint stiffness, which reflects a joint’s ability to resist deformation under external forces, is also crucial for knee stability [52,66]. These findings suggest that HL athletes have superior knee joint stability, which may reduce their susceptibility to injuries. To improve knee joint stability in LL athletes, strength training and movement retraining should be emphasized. Exercises that promote greater knee flexion during landing, such as deep squats, single-leg squats, and step-down drills, can enhance shock absorption capacity and reduce anterior tibial shear forces. Additionally, muscular training, including plyometric drills with proper landing mechanics (e.g., maintaining knee alignment and controlled deceleration), may help optimize landing strategies [67,68]. Resistance training focused on the quadriceps, hamstrings, and gluteal muscles can contribute to improved joint stability and load distribution during high-impact movements.

LL and HL athletes performed a 360° straight-body jump in our experiment. During the landing phase, gravitational potential energy is converted into kinetic energy, with the impact force primarily absorbed by the lower limb muscles and skeleton. Compared to LL athletes, HL athletes exhibited higher muscle force and activation, particularly in the MG, LG, and TA muscles. Devita and Turbanski have demonstrated that increasing calf muscle strength contributes to better energy dissipation [69,70], as the more energy absorbed by the muscles, the less force is transferred to the bones. Furthermore, during the landing phase, impact forces are transferred from distal to proximal: transmission from the ankle joint to the knee and to the hip joint [43,44]. Our results demonstrate that HL athletes exhibited greater energy dissipation at the ankle and throughout the lower limb, resulting in relatively smaller impact forces at the knee joint. We hypothesize that this difference stems from HL athletes having a greater initial contact angle at the ankle joint. Earlier studies have indicated that a greater initial contact angle at the ankle joint during landing enhances energy dissipation [43]. In contrast, LL athletes experienced higher impact forces at the ankle joint and exhibited significantly larger ankle inversion and internal rotation angles, suggesting a higher risk of ankle inversion injuries. To reduce the risk of ankle inversion injuries, ankle stability training should be a key component of injury prevention programs. Balance board drills, single-leg stance training, and dynamic stability exercises (e.g., lateral hops and perturbation training) can enhance proprioception and neuromuscular control, which are crucial for maintaining joint stability during landing [71,72]. Additionally, strengthening the calf muscles, particularly the gastrocnemius and soleus, can improve the ankle’s ability to dissipate impact forces. Increased muscle strength in this region not only enhances energy absorption but also reduces excessive ankle inversion and internal rotation, both of which are linked to a higher risk of lateral ankle sprains. These methods can enhance dynamic ankle stability and reduce injury risk in high-impact movements.

Besides, we used EMG to verify the OpenSim model, which showed a significant correlation between the simulated muscle activation and actual EMG data. This correlation enabled us to differentiate between the agonist and antagonist muscles and to calculate muscle coactivation [73]. The posterior calf muscles act as agonists for plantarflexion, while the TA is the agonist for dorsiflexion. Coactivation of these muscles is essential for stabilizing the ankle joint, controlling its displacement, and absorbing impact forces during landing [74,75]. Our results showed that LL athletes exhibited lower muscle coactivation ratios around the ankle joint (e.g., LG/TA, SL/TA, and MG/TA), resulting in lower ankle joint stability. Previous studies by Pan and Yang have emphasized that lower extremity joint stability largely depends on muscle coactivation [76,77]. In contrast, HL athletes demonstrated higher coactivation ratios around the ankle joint, along with a smaller dorsiflexion angle and a larger plantarflexion moment. We hypothesize that higher coactivation ratios generate a stronger plantarflexion moment to counter excessive dorsiflexion, as evidenced by the greater dorsiflexion angle and moment observed in LL athletes. Additionally, HL athletes showed smaller BF/RF and BF/VL ratios around the knee joint. We suggest that HL athletes have more precise control over muscle contraction and relaxation, optimizing coordination between BF and RF, as well as between BF and VL. Specifically, RF and VL gradually relax during knee flexion, while BF actively drives the knee into flexion. This coordination results in more stable and efficient knee flexion. The greater knee flexion angle observed in HL athletes further supports this finding. Therefore, it can be concluded that HL athletes exhibit better stability at both the ankle and knee joints compared to LL athletes.

There are several limitations to this study. First, restricting the study to only male competitive aerobics athletes may reduce the generalizability of the findings. Physiological, anatomical, and performance differences between males and females (e.g., variations in skeletal structure, muscle strength, and hormone levels) may lead to different lower limb biomechanical properties. Future research should include female participants and athletes from different institutions or professional settings to provide a broader understanding of the differences in athletic performance and biomechanics between genders. Second, all participants were from a single university, which ensures a controlled training environment but may limit the generalizability of the findings to athletes from other institutions, professional settings, or different training systems. Future research should expand participant recruitment to include athletes from multiple universities, professional teams, and different competitive levels to enhance external validity. Furthermore, the specific jump task used in this study (e.g., 360° straight-body jump) may not fully represent various movements that athletes use in competitions. Real-world competitions involve more diverse and complex movements, including different types of jumps and rotations. Future research should incorporate more game-specific movements or analyze competition footage to design more varied and realistic experimental tasks, thereby increasing the external validity and practical relevance of the findings. To conclude, this study offers important insights regarding lower limb biomechanical differences between athletes of different levels during rotational jump landings, but the identified limitations suggest avenues for further research.

## 5. Conclusions

In summary, this research explored the differences in lower extremity biomechanical characteristics during rotational jump landings between HL and LL athletes. Compared to HL athletes, LL athletes exhibit poorer stability at the ankle and knee joint. LL athletes are more prone to ACL injuries and ankle inversion injuries during the landing phase of the rotational jump. To reduce these risks, LL athletes should adopt a landing technique that increases the flexion angle of the knee and hip joints, as well as the ankle plantarflexion angle. Future research will focus on more challenging movements to further investigate the underlying biomechanical mechanisms in competitive aerobics.

## Data Availability

The data are not publicly available due to privacy or restrictions on ethics.

## Supplementary Materials

The following supporting information can be downloaded at: https://www.mdpi.com/article/10.3390/bioengineering12030220/s1, Table S1: The peak angle, moment and power of the ankle, knee, and hip joint across three planes (Sagittal plane, Frontal plane, and Transverse plane) between LL and HL athletes (Mean ± SD); Table S2: Comparison of mean energy dissipation and contribution to total energy dissipation in the sagittal plane and (Means ± SD) between LL and HL athletes during landing phase; Table S3: Comparison of changes in peak muscle activation and force between LL and HL athletes during landing phase.
