# Supplementary material for: Exploring Lower Limb Biomechanical Differences in Competitive Aerobics Athletes of Different Ability Levels During Rotational Jump Landings
Source: Bioengineering (Basel). 2025 Feb 21;12(3):220. doi: 10.3390/bioengineering12030220 (PMC11939504; doi:10.3390/bioengineering12030220)
Supplement: Supplementary file 1 [file bioengineering-12-00220-s001.zip › bioengineering-3444074-supplementary.pdf]

Supplemental Material

Exploring Lower Limb Biomechanical Differences in Competitive Aerobics Athletes of Different Ability Levels during Rotational Jump Landing

This Supporting Material file includes:

Results

Table 1 to Table 3

Table 1. The peak angle, moment and power of the ankle, knee, and hip joint across three planes (Sagittal plane, Frontal plane, and Transverse plane) between LL and HL athletes (Mean ± SD).

Parameters		Peak-Value	LL Mean ± SD	HL Mean ± SD	Cohen′s d	p-Value
Ankle	Angle (°)	Dorsiflexion	26.5 ± 4.26	22.31 ± 3.73	1.05	**<0.001***
		Plantarflexion	-11.37 ± 9.34	-15.85 ± 12.13	0.41	0.095
		Eversion	-0.66 ± 2.05	4.34 ± 2.65	2.11	**<0.001***
		Inversion	-10.88 ± 2.19	-9.93 ± 1.63	0.49	0.132
		ER	0.07 ± 1.61	1.42 ± 2.85	0.58	**0.04***
		IR	-9.98 ±1.12	-8.57 ± 1.26	1.18	**<0.001***
	Moment (Nm/kg)	Dorsiflexion	-0.42 ± 0.36	-0.87 ± 0.41	0.95	**<0.001***
		Plantarflexion	-3.31 ± 0.68	-3.8 ± 0.65	0.32	**0.016***
		Eversion	0.13 ± 0.15	-0.05 ± 0.12	1.33	**<0.001***
		Inversion	-1.08 ± 0.42	-0.97 ± 0.38	0.27	0.364
		ER	-0.16 ± 0.09	-0.01 ± 0.1	1.58	**<0.001***
		IR	-0.66 ± 0.15	-0.63 ± 0.19	0.18	0.509
	Power (W)	Dorsiflexion	1.64 ± 1.55	1.74 ± 1.11	0.07	0.789
		Plantarflexion	-23.45 ± 8.28	-25.91 ± 11.67	0.24	0.377
		Eversion	2.17 ± 2.3	2.29 ± 1.55	0.06	0.865
		Inversion	-0.53 ± 0.38	-0.4 ± 0.6	0.26	0.438
		ER	2.56 ± 0.24	3.12 ± 0.67	1.11	0.056
		IR	-0.36 ± 0.1	-0.41 ± 0.21	0.3	0.587
Knee	Angle (°)	Flexion	-76.82 ± 6	-88.46 ± 5.48	2.03	**<0.001***
		Extension	-22.53 ± 5.61	-18.39 ± 8.08	0.60	**0.026***
		Adduction	1.07 ± 1.35	1.38 ± 0.39	0.19	0.339
		Abduction	6.55 ± 0.79	5.64 ± 0.64	1.27	**<0.001**

		ER	-3.32 ± 3.53	-4.14 ± 4.1	0.21	0.411
		IR	10.79 ± 2.37	4.05 ± 2.85	2.57	**<0.001***
	Moment (Nm/kg)	Flexion	3.14 ± 1.09	4.3 ± 0.9	1.16	**<0.001***
		Extension	0.06 ± 0.41	0.25 ± 0.4	0.47	0.106
		Abduction	-0.99 ± 0.58	-1.14 ± 0.47	0.28	0.429
		Adduction	0.31 ± 0.37	0.28 ± 0.3	0.09	0.822
		ER	-0.06 ± 0.1	-0.04 ± 0.1	0.2	0.404
		IR	1.21 ± 0.39	1.32 ± 0.36	0.29	0.36
	Power (W)	Flexion	-29.24 ± 12.04	-30.63 ± 10.46	0.12	0.695
		Extension	1.38 ± 0.84	1.85 ± 1.1	0.48	0.138
		Abduction	0.35 ± 0.56	0.48 ± 0.29	0.29	0.562
		Adduction	-2.52 ± 1.25	-1.95 ± 0.95	0.51	0.274
		ER	-1.37 ± 0.49	-1.08 ± 0.58	0.54	0.106
		IR	0.2 ± 0.13	0.19 ± 0.13	0.08	0.769
	Angle (°)	Flexion	55.54 ± 4.3	60.66 ± 4.27	1.19	**<0.001***
		Extension	25.34 ± 5.91	24.54 ± 7.23	0.12	**0.627***
		Abduction	19.98 ± 1.16	19.27 ± 1.75	0.48	0.168
		Adduction	9.03 ± 2.59	10.93 ± 2.96	0.68	0.058
		ER	6.54 ± 1.86	7.36 ± 2.69	0.35	0.284
		IR	14.07 ± 0.96	16.09 ± 2.25	1.17	**<0.001**
	Moment (Nm/kg)	Flexion	-3.69 ± 1.78	-4.52 ± 1.79	0.46	0.106
		Extension	0.38 ± 0.59	1.06 ± 0.68	1.07	**<0.001***
Hip		Abduction	0.4 ± 0.5	0.84 ± 0.69	0.73	**0.024***
		Adduction	-1.84 ± 1.01	-2.13 ± 0.99	0.29	0.358
		IR	-0.32 ± 0.3	-0.22 ± 0.19	0.4	0.25
		ER	0.37 ± 0.22	0.52 ± 0.15	0.8	**0.018***
	Power (W)	Flexion	-28.81 ± 9.36	-23.49 ± 6.53	0.66	0.077
		Extension	0.92 ± 0.56	2.33 ± 1.69	1.12	**0.003***
		Abduction	0.43 ± 0.44	0.73 ± 0.55	0.6	0.188
		Adduction	-3 ± 0.55	-4.17 ± 0.4	2.43	**<0.001***
		ER	0.29 ± 0.19	0.17 ± 0.12	0.76	0.098
		IR	-0.75 ± 0.25	-0.75 ± 0.11	0	0.979

Note: °: Degrees; Nm/kg: Newton meters per kilogram; W: Watt; SD: Standard deviation. "*" means significance with $p < 0.05$.

Table 2: Comparison of mean energy dissipation and contribution to total energy dissipation in the sagittal plane and (Means ± SD) between LL and HL athletes during landing phase.

Biomechanical variables		LL Mean ± SD	HL Mean ± SD	Cohen's d	p-Value
Ankle	Mean energy dissipation (J/kg)	-0.82 ± 0.37	-1.39 ± 0.96	0.78	**0.011***
	Contribution to total energy dissipation (%)	18.78 ± 8.47	28.38 ± 20	0.63	**0.033***
Knee	Mean energy dissipation (J/kg)	-2.06 ± 0.6	-2.49 ± 0.61	0.71	0.196
	Contribution to total energy dissipation (%)	47.01 ± 25.99	50.81 ± 19.06	0.17	0.593
Hip	Mean energy dissipation (J/kg)	-1.5 ± 0.6	-1.02 ± 0.36	0.97	**0.029***
	Contribution to total energy dissipation (%)	34.21 ± 14.38	20.81 ± 7.49	1.17	**0.009***
	Total energy dissipation (J/kg)	-4.39 ± 0.51	-4.9 ± 0.62	0.90	**0.003***

Note: J/kg: Joules per kilogram. "*" means significance with $p < 0.05$.

Table 3: Comparison of changes in peak muscle activation and force between LL and HL athletes during landing phase.

Peak-Value	Muscles	LL Mean ± SD	HL Mean ± SD	Cohen's d	p-Value
Muscle Activation (&MVC)	RF	0.73 ± 0.06	0.81 ± 0.06	1.33	**0.002***
	VM	0.66 ± 0.03	0.78 ± 0.06	2.53	**< 0.001***
	BF	0.7 ± 0.03	0.79 ± 0.03	3	**< 0.001***
	MG	0.51 ± 0.08	0.57 ± 0.1	0.66	0.108
	LG	0.53 ± 0.04	0.56 ± 0.07	0.53	0.123
	VL	0.56 ± 0.06	0.62 ± 0.05	1.09	**0.003***
	SL	0.59 ± 0.05	0.68 ± 0.06	1.63	**< 0.001***
	TA	0.63 ± 0.04	0.69 ± 0.06	1.18	**0.015***
Muscle Force (BW)	RF	3.46 ± 0.89	4.36 ± 1.28	0.82	**0.011***
	VM	1.48 ± 0.37	1.61 ± 0.29	0.39	0.138
	BF	2.18 ± 0.77	2.88 ± 0.58	1.03	**0.005***
	MG	2.02 ± 0.41	2.41 ± 0.53	0.82	**0.014***
	LG	2.12 ± 0.34	2.58 ± 0.33	1.37	**< 0.001***
	VL	2.27 ± 0.55	2.55 ± 0.47	0.55	**0.037***
	SL	1.45 ± 0.2	1.52 ± 0.23	0.33	0.368
	TA	2.14 ± 0.55	2.38 ± 0.56	0.43	0.114

Note: "*" indicates a significant difference between LL and HL athletes during landing phase ($p < 0.05$); BW: Body Weight.
